# Modeling the Potential Invasion Risk of *Ageratina adenophora* in China From an Ecological Suitability Perspective

**DOI:** 10.1002/ece3.72392

**Published:** 2025-10-22

**Authors:** Xiaolan Xie, Tian Ma, Yu Chen, Jun Zhuo, Shuai Chen, Tingting Kang, Mengmeng Hao, Fangyu Ding, Dong Jiang

**Affiliations:** ^1^ School of Geography & Environmental Science Guizhou Normal University Guiyang China; ^2^ Yale Institute for Biospheric Studies Yale University New Haven Connecticut USA; ^3^ School of the Environment Yale University New Haven Connecticut USA; ^4^ Southwest Computer co., LTD Chongqing China; ^5^ Institute of Geographic Sciences and Natural Resources Research Chinese Academy of Sciences Beijing China; ^6^ College of Resources and Environment University of Chinese Academy of Sciences Beijing China; ^7^ School of Urban Planning and Design Peking University, Shenzhen Graduate School Shenzhen China

**Keywords:** *Ageratina Adenophora*, climate change, future prediction, human activity, invasion risk, random forest

## Abstract

Invasive species like *Ageratina adenophora* significantly threaten biodiversity, ecosystem stability, and economic resources in China. This study assesses the current and future potential invasion risk of *A. adenophora* across China, focusing on the provinces of Yunnan, Sichuan, Guizhou, and Guangxi—areas identified as highly susceptible to invasion. Utilizing both climate and human activity data, our results reveal that population density and temperature seasonality are dominant national‐scale factors that affect the invasion risk of *A. adenophora* , while regional drivers exhibit significant variation. Projections show a northward and altitudinal shift in invasion risk by the 2060s, highlighting new potential invasion hotspots driven by future changes in climate and human activities. These findings underscore the importance of region‐specific management strategies and establish a foundation for adaptive measures to mitigate the ecological and economic impacts of *A. adenophora* .

## Graphical abstract

Comprehensive analysis of *A. adenophora* invasion risk in China under CMIP6 scenarios. RF model used to simulate current and future distributions at national and provincial levels. Factors influencing invasion risk exhibit significant variation at the regional level. Projections indicate northward and altitudinal risk shifts by 2060s in key provinces.

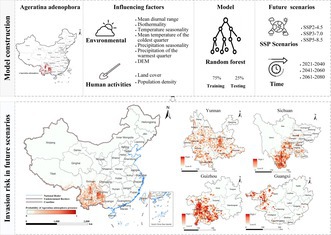

## 1 Introduction

Species invasions, where non‐native organisms spread in new ecosystems, cause biodiversity loss, habitat disruption, and economic costs (Blackburn et al. 2011; Early et al. 2016; Simberloff et al. 2013; Vilà et al. 2010). In China, the spread of *Ageratina adenophora* , which was introduced from the Americas in the 1940s, illustrates the scale of impact invasives can have on native ecosystems (Wang and Wang 2006). *A. adenophora* is widespread across Yunnan and rapidly advancing through other regions. It has adapted well to the subtropical monsoon climate of southern China, which closely resembles its native habitat (Li, Song, et al. 2022; Xian et al. 2022). This adaptability has facilitated its aggressive spread, resulting in the displacement of native plant species (Fu et al. 2018), decreased agricultural productivity (Poudel et al. 2019), and altered soil composition (Yang et al. 2024), thereby significantly threatening local biodiversity and ecosystem stability. These disruptions highlight the urgent need to understand the distribution range of *A. adenophora* and the factors driving its spread, thereby enabling the development of more targeted management strategies to prevent its invasion into new potentially suitable areas.

Climate change has reshaped species invasions by altering habitats and species behaviors globally and regionally (Bellard et al. 2018; Rodrigues et al. 2024). For example, rising temperatures and changing precipitation patterns extend the range of habitable zones for numerous invasive species, thus facilitating their spread and intensifying ecological impacts (Bradley et al. 2010; Seebens et al. 2021). These environmental shifts allow invasive plants to thrive in new areas that were previously unsuitable for survival (Gong et al. 2020). According to the Sixth Assessment Report of the Intergovernmental Panel on Climate Change (IPCC), these climatic changes are expected to continue and intensify, further modifying ecosystems (IPCC 2021). Such changes are likely to create more favorable conditions for invasive species, altering their distribution patterns and increasing the vulnerability of certain regions to invasions (Tu et al. 2021). Alongside climate impacts, human activities, such as land conversion and agricultural expansion, also significantly influence species invasions (Pysek et al. 2010). For instance, land use changes disrupt ecosystems, causing habitat fragmentation that diminishes native species diversity and abundance (Haddad et al. 2015; Raveloaritiana et al. 2024). These activities create opportunities for invasive species to spread and establish new territories (Chen et al. 2021).

Based on these factors, an increasing number of studies are utilizing species distribution models to predict the potential invasive risk of *A. adenophora* . For example, Gu et al. (Gu et al. 2021) utilized multiple Representative Concentration Pathways (RCPs) to predict the possible future distribution of *A. adenophora* across various climate scenarios on a global scale, providing valuable insight into its worldwide spread. Moreover, several studies have examined the species' future invasion in regions such as South Africa, India, and Nepal, demonstrating its adaptability to diverse environmental conditions (Chaudhary et al. 2021; Poudel et al. 2020; Tererai and Wood 2014; Verma et al. 2023). Given the considerable threat posed by *A. adenophora* in China, numerous researchers have also focused on modeling its distribution in China (Fang et al. 2021; Tu et al. 2021; Xian et al. 2022, 2023). These studies have advanced our understanding of the species' ecological adaptability and the factors influencing its spread, providing a foundation for developing predictive models and management strategies.

However, existing studies primarily focus on climate factors while underrepresenting human influences such as land use changes and population expansion, which significantly alter habitats and create favorable conditions for invasive species. The omission of these human‐induced factors may limit the accuracy of predictive models (Early and Sax 2014; Essl et al. 2011). Additionally, most studies continue to rely on Coupled Model Intercomparison Project–Phase 5 (CMIP5) climate scenarios despite the release of the updated CMIP6 framework, which offers higher climate sensitivity and a broader range of scenarios (Eyring et al. 2016; Meehl et al. 2020). Furthermore, much of the current research considers China a homogeneous region, overlooking significant regional variations. Provinces such as Yunnan, Sichuan, Guizhou, and Guangxi, where *A. adenophora* is most prevalent, display distinct local climates and human activity patterns that influence invasion dynamics (Gu et al. 2021; Zhang et al. 2022). These gaps emphasize the importance of region‐specific models that incorporate both environmental and anthropogenic factors to support more targeted management strategies.

In this study, we provide a comprehensive analysis of the invasion risk of *A. adenophora* in China, integrating both climatic and human‐induced factors using the CMIP6 climate framework. We initially used the random forest (RF) model to simulate the current distribution of *A. adenophora* across China and in four key provinces—Yunnan, Sichuan, Guizhou, and Guangxi. We then examined the influence of various environmental and anthropogenic factors to evaluate their relative importance in driving the species' invasion across these regions. Finally, we used CMIP6 scenarios to project the potential invasive risk of *A. adenophora* for the periods 2021–2040, 2041–2060, and 2061–2080 under three Shared Socioeconomic Pathways (SSPs: 245, 370, and 585). This approach enables an assessment of how climate change and human activities may collectively affect the invasion dynamics of *A. adenophora* , providing critical insights for targeted management strategies. By enhancing our understanding of region‐specific invasion patterns, this study contributes to the foundation of management practices to mitigate the ecological and economic impacts of *A. adenophora* in areas most vulnerable to future invasions.

## 2 Materials and Methods

### 2.1 Occurrence Data of *Ageratina adenophora*

**FIGURE 1:**
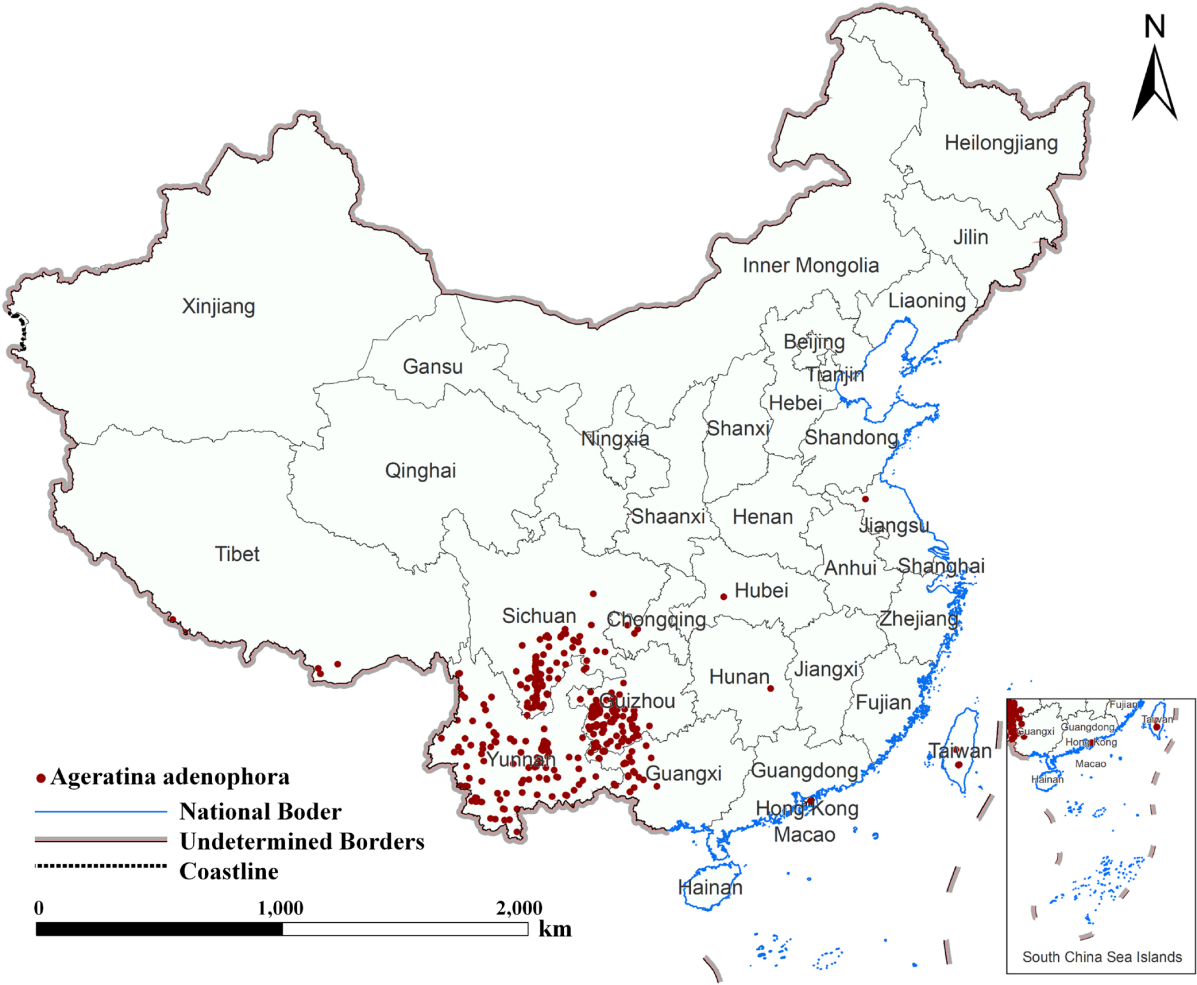
Occurrence records of *A. adenophora* in China.

The occurrence records of *A. adenophora* were collected from the Global Biodiversity Information Facility (GBIF), the Chinese Virtual Herbarium (CVH, http://www.cvh.ac.cn/), and the National Specimen Information Infrastructure (NSII, http://www.nsii.org.cn/). Records lacking precise location information were excluded to maintain data quality. After careful verification and removal of duplicate entries, 368 occurrence records were retained for analysis, as illustrated in Figure 1. The records are primarily concentrated in southwestern China, particularly in Yunnan, Sichuan, Guizhou, and Guangxi provinces, with Yunnan having the highest density. Occurrence points extend into adjacent provinces/regions, including Chongqing, Hunan, and Guangdong.

### 2.2 Environmental and Human Activities Data

Environmental variables are essential in determining species distributions (Fick and Hijmans 2017). Nineteen bioclimatic variables are commonly recognized for their significance in species distribution modeling (Seebens et al. 2021). Elevation, an essential factor for many plant species, was included as a predictor for the distribution of invasive species (Li, Zheng, et al. 2022). The bioclimatic variables and digital elevation model (DEM) data were sourced from the WorldClim dataset (www.worldclim.org).

Human activities, such as land use changes and population density, also contribute significantly to the spreading and establishing of invasive species like *A. adenophora* (Seebens et al. 2021). To account for these impacts in the model, population density and land cover data were incorporated as proxies for human influence. Population density reflects the intensity of human presence and habitat pressure, with more remarkable densities typically associated with greater habitat alteration (Venter et al. 2016). Land cover data capture the extent and nature of land cover changes, such as converting natural ecosystems into agricultural or urban areas, directly influencing habitat suitability for invasive species (Pysek et al. 2020). Population density data were retrieved from the Socioeconomic Data and Applications Center (SEDAC, http://sedac.ciesin.columbia.edu), and land cover data from the Land‐Use Harmonization project (LUH2, https://luh.umd.edu/). Integrating these datasets with environmental variables provides us with a comprehensive understanding of how human activities and environmental factors collectively influence the invasion of *A. adenophora* .

To ensure predictor independence and mitigate multicollinearity, Pearson's correlation analysis was applied to 22 candidate variables (Table S1) derived from environmental and human activity data (Dormann et al. 2013). Predictors with correlation coefficients exceeding 0.75 were subsequently excluded (Tu et al. 2021). Following this filtering process, nine variables were retained for analysis: mean diurnal range (Bio2), isothermality (Bio3), temperature seasonality (Bio4), mean temperature of the coldest quarter (Bio11), precipitation seasonality (Bio15), precipitation of the warmest quarter (Bio19), DEM, land cover, and population density.

To project future invasion of *A. adenophora* under changing climate and human activity conditions, the Beijing Climate Center Climate System Model (BCC‐CSM), developed by the National Climate Center of the China Meteorological Administration, was selected for its suitability in climate change studies within China (Wu et al. 2019). Based on the 2021 IPCC Sixth Assessment Report (AR6) (IPCC 2021), three future periods were selected: 2021–2040, 2041–2060, and 2061–2080. Simulations were conducted under three shared socioeconomic pathways (SSPs): SSP245 (moderate scenario), SSP370 (fragmented scenario), and SSP585 (pessimistic scenario). All variable layers used in the analysis had a spatial resolution of 10 × 10 km.

### 2.3 Model Construction

The RF model was chosen for this study due to its ability to model non‐linear relationships between variables (Breiman 2001). Its ensemble learning approach, which averages predictions from multiple decision trees, helps reduce overfitting and improves model robustness (Cutler et al. 2007; Prasad et al. 2006). These characteristics make RF particularly well‐suited for modeling the distribution of invasive species like *A. adenophora* .

The RF models were built using the R programming language (version 4.3.2) and the randomForest package. The model was trained using 75% of the occurrence data, with the remaining 25% reserved for testing. 1000 decision trees (ntree = 1000) were generated, while all other parameters were kept at their default settings.

As previously described, the model input consisted of nine predictors. The response variable was the presence or absence of *A. adenophora* based on observed occurrence data. The RF model produced a probability score between 0 and 1 for each location, where higher values indicated greater habitat suitability for *A. adenophora* . A value closer to 1 suggests highly favorable conditions, while lower values indicate less suitable habitats.

### 2.4 Model Evaluation and Outputs

RF model performance was assessed through receiver operating characteristic (ROC) curves and area under the curve (AUC) metrics, a widely accepted metric for assessing binary classification models (Lobo et al. 2008; Provost and Fawcett 2001). ROC‐AUC provides a comprehensive measure of model accuracy across all classification thresholds, with values ranging from 0 to 1 (Fawcett 2006). Higher ROC‐AUC scores indicate better model performance. This study used ROC‐AUC to evaluate the model's ability to distinguish between suitable and unsuitable habitats for *A. adenophora* . A high ROC‐AUC value suggests that the model is highly effective in predicting the potential invasive risk for the species.

To better understand the impact of each predictor variable on the model's predictions, variable importance scores were calculated within the RF model to evaluate each variable's contribution (Breiman 2001). Variables with higher importance scores significantly influenced the potential invasion risk of *A. adenophora* . Furthermore, partial dependence plots (PDPs) were generated to explore the effects of individual predictors on the predicted probability of species invasion. PDPs illustrate how variations in one predictor, while holding others constant, affect habitat suitability (Friedman 2001). These plots offer deeper insights into the role of each environmental or human activity variable in determining the potential invasion of *A. adenophora* .

## 3 Result

### 3.1 Current Distribution of *A. adenophora* in China

**FIGURE 2:**
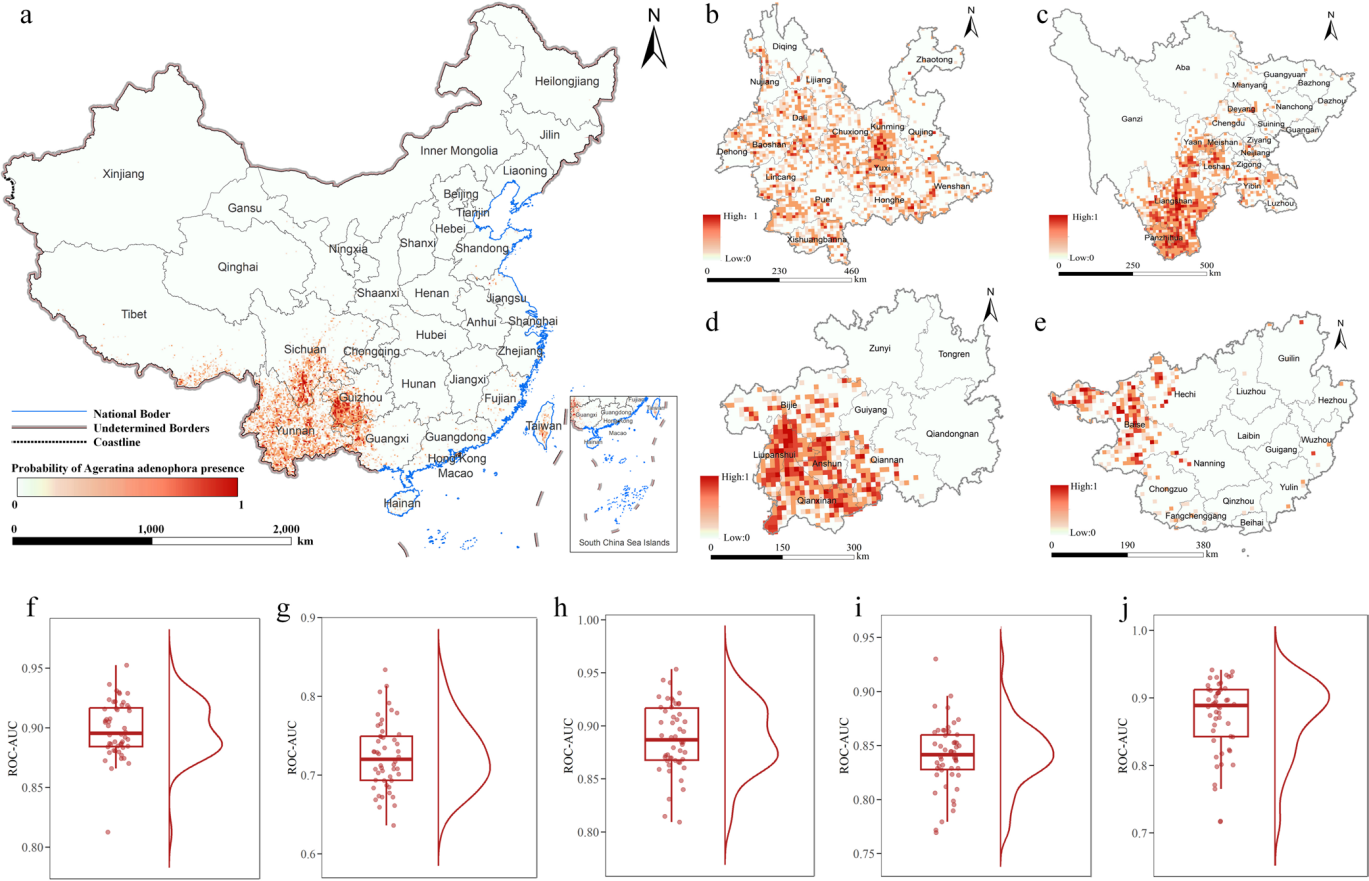
Simulated distribution and ROC‐AUC for *A. adenophora* in China and critical provinces. Panels a‐e display the simulated invasion risk of *A. adenophora* in China and the four key provinces. Panels f‐j show the boxplots of 50 ROC‐AUC values for the national model (China) and the four provinces (Yunnan, Sichuan, Guizhou, and Guangxi).

Based on the occurrence data of *A. adenophora* , we applied the RF model to simulate its potential invasive risk across China, focusing specifically on the four key provinces of Yunnan, Sichuan, Guizhou, and Guangxi (Figure 2). Results suggest that the RF model performs well in predicting the invasion of *A. adenophora* at both the national and provincial levels, though the slightly lower ROC‐AUC for Yunnan may indicate challenges in capturing the species' distribution patterns in that region.

The simulation results indicate that the potential invasive risk of *A. adenophora* in China extends beyond its currently known presence, with a prominent concentration in the southern and southwestern regions (Figure 2a). Guangxi, Guizhou, Sichuan, and Yunnan are predicted to have the highest risk of invasion, consistent with observed patterns. At the provincial level, *A. adenophora* shows a widespread but fragmented distribution in Yunnan, indicating a patchy spread of the species (Figure 2b). In Sichuan, the model predicts the invasion risk primarily concentrated in the southwestern regions, including Panzhihua and Liangshan (Figure 2c). In Guizhou, the invasion risk is focused mainly in the western and southwestern areas, particularly in Bijie, Liupanshui, Anshun, and parts of Qianxinan (Figure 2d). In Guangxi, the invasion risk is primarily concentrated in the northwestern region, particularly around Baise and Hechi (Figure 2e). These provincial insights help clarify how *A. adenophora* spreads differently across regions.

### 3.2 Main Determinants of the Invasion for *A. adenophora*

**FIGURE 3:**
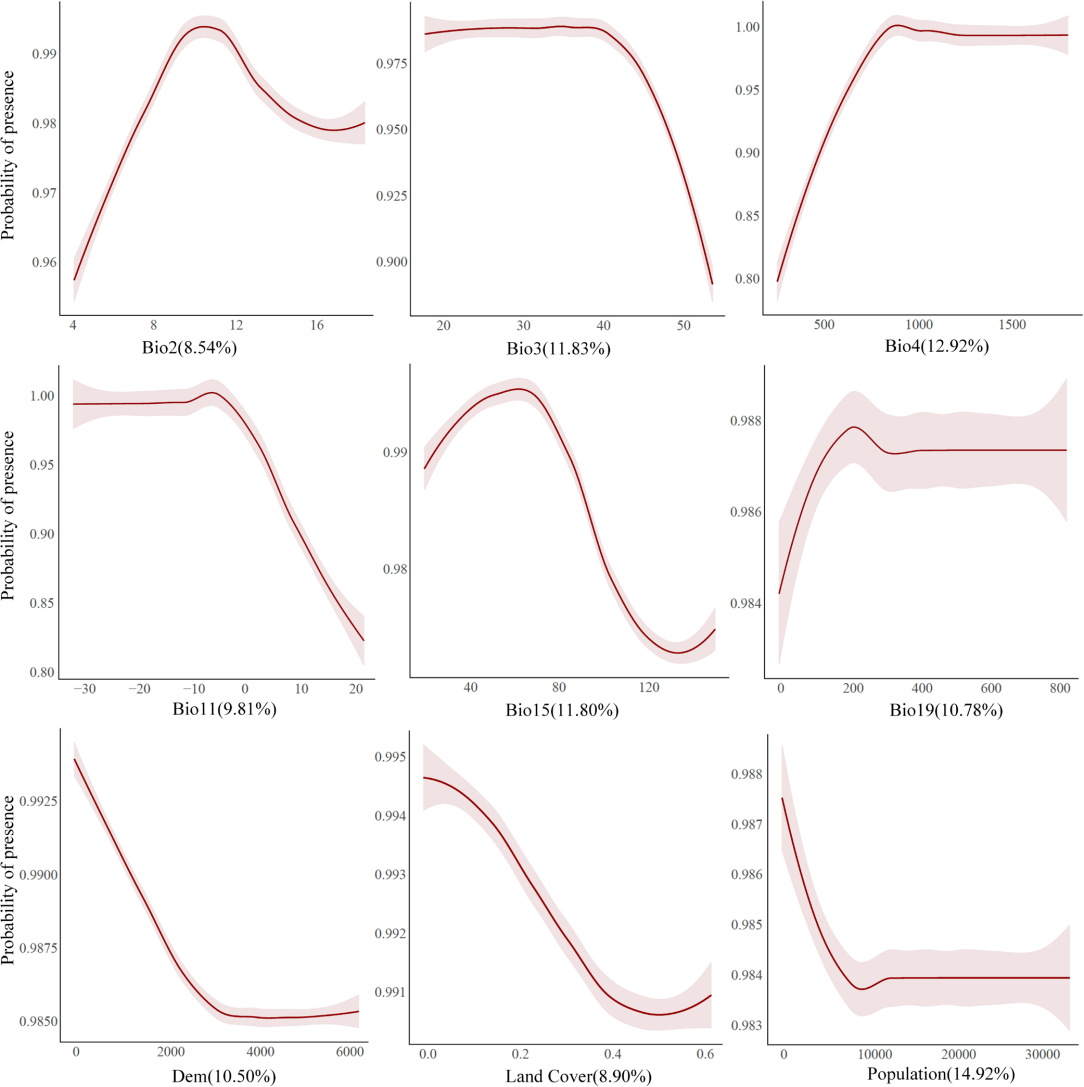
Partial dependence plots of factors and the probability of invasion risk for *A. adenophora* . Each panel represents the relationship between a specific factor and the likelihood of the invasion of the species. The red line indicates the estimated effect of the variable on habitat suitability, while the shaded pink areas represent the confidence intervals. The percentage in parentheses represents the contribution of each variable to the overall model.

A range of factors influences the invasion of *A. adenophora* across both national and provincial scales. Population density is the most impactful factor at the national level (Figure 3, Table S2), contributing 14.92%. Temperature‐related variables such as temperature seasonality (Bio4) and isothermality (Bio3) also play important roles, contributing 12.92% and 11.83%, respectively.

At the provincial level, the importance of factors varies. In Yunnan (Table S3), population density (12.88%) and cropland (12.29%) are significant, alongside climatic factors like mean diurnal range (Bio2, 11.21%) and precipitation of the coldest quarter (Bio19, 11.77%), which are also crucial. In Sichuan (Table S4), temperature seasonality (Bio4, 17.60%) is the dominant factor, with precipitation seasonality (Bio15, 11.13%) and mean temperature of the coldest quarter (Bio11, 11.22%) also contributing. In Guizhou (Table S5), precipitation patterns are critical, with precipitation seasonality (Bio15, 13.15%) and precipitation of the coldest quarter (Bio19, 13.12%) being the most vital contributors, while population density (4.77%) has a comparatively minor impact. In Guangxi (Table S6), temperature seasonality (Bio4, 14.45%) is the main factor, followed by precipitation of the coldest quarter (Bio19, 12.83%) and mean diurnal range (Bio2, 12.64%).

Partial dependence plots were generated to evaluate the relationship between each factor and the invasion risk of *A. adenophora* . At the national scale (Figure 3), the results show that the invasion risk of *A. adenophora* generally increases with moderate values of annual temperature range (Bio2), temperature seasonality (Bio4), and precipitation seasonality (Bio15) but decreases at extreme values. Invasion risk decreases as elevation increases, and population density initially reduces suitability before stabilizing. The influence of land cover and minimum temperature (Bio11) also suggests that certain conditions, like extremely low temperatures, are less favorable for the growth of *A. adenophora* . Overall, moderate climatic conditions support its habitat suitability. At the provincial scale, the response of most factors aligns with the national trends, though some factors show notable differences. These differences are detailed in Figures S1–S4.

### 3.3 Projected Invasion Risk of *A. adenophora* Under Future Climate and Human Activity Changes

The predicted invasion risk of *A. adenophora* in China, as well as in the critical provinces of Yunnan, Sichuan, Guizhou, and Guangxi, is expected to shift significantly under future climate and human activity changes during the periods 2020s (2021–2040), 2040s (2041–2060), and 2060s (2061–2080), based on projections across three scenarios (SSP2‐4.5, SSP3‐7.0, and SSP5‐8.5). The predictions highlight both the potential invasion risk and the spatiotemporal changes in these regions, reflecting the influence of climate change and human activity change on invasion risk.

**FIGURE 4:**
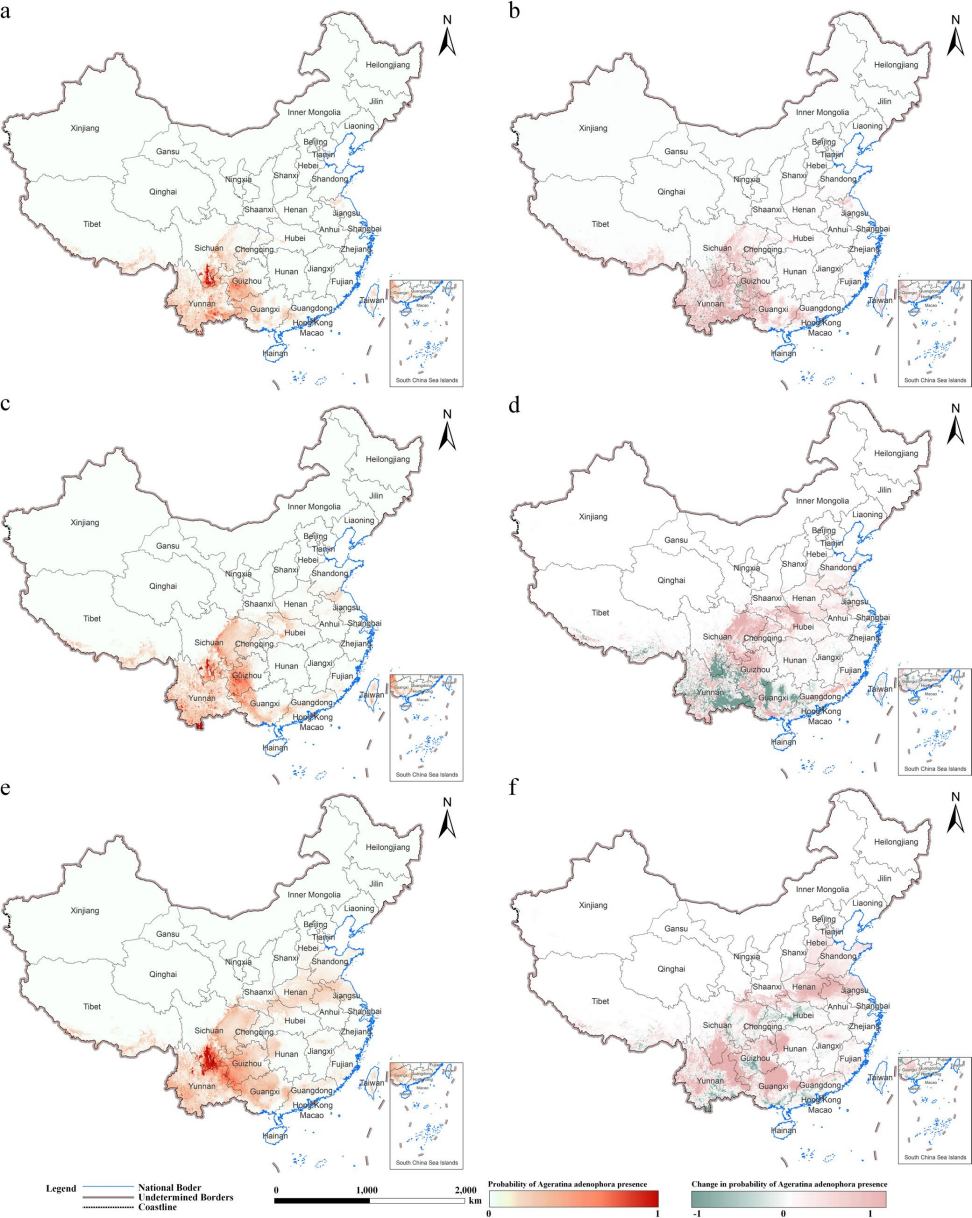
Predicted invasion risk and changes for *A. adenophora* under SSP 3–7.0 climate and human activity scenario (2021–2080). The left column (a, c, e) shows the predicted probability of invasion risk for the species in each period, with deeper red colors indicating higher probabilities. The right column (b, d, f) shows the changes in predicted invasion risk: (b) represents changes from the current period to 2021–2040, (d) shows changes from 2021–2040 to 2041–2060, and (f) indicates changes from 2041–2060 to 2061–2080.

Under the SSP3‐7.0 scenario, short‐term projections for the 2020s in China indicate that the southern and southwestern regions, especially Yunnan, Guangxi, and parts of Sichuan, exhibit the highest invasion risk for *A. adenophora* (Figure 4a). The species is projected to expand into previously only marginally suitable areas. Compared to current climate conditions, these regions show substantial increases in invasion risk (Figure 4b). Mid‐term projections for the 2040s suggest that the invasion risk for *A. adenophora* will extend northward and into higher elevations (Figure 4c). While the southwestern regions, including Yunnan and Guizhou, remain consistently suitable, central China—particularly in mountainous areas—increases invasion risk (Figure 4d). However, some southern areas of China (e.g., Kunming City, Guiyang City) may begin to experience slight decreases in invasion risk. Long‐term projections for the 2060s show continued expansion of invasion risk across central and western China (Figure 4e). Northern Sichuan and other parts of central China are projected to see further increases in invasion risk (Figure 4f). Yunnan remains a core area of high invasion risk; some southern regions may see declines. While the prediction results are generally consistent across all SSP scenarios, the high invasion risk areas are significantly larger under SSP5‐8.5 compared to SSP2‐4.5 and SSP3‐7.0, and this difference remains consistent over time (Figures S5 and S6).

**FIGURE 5:**
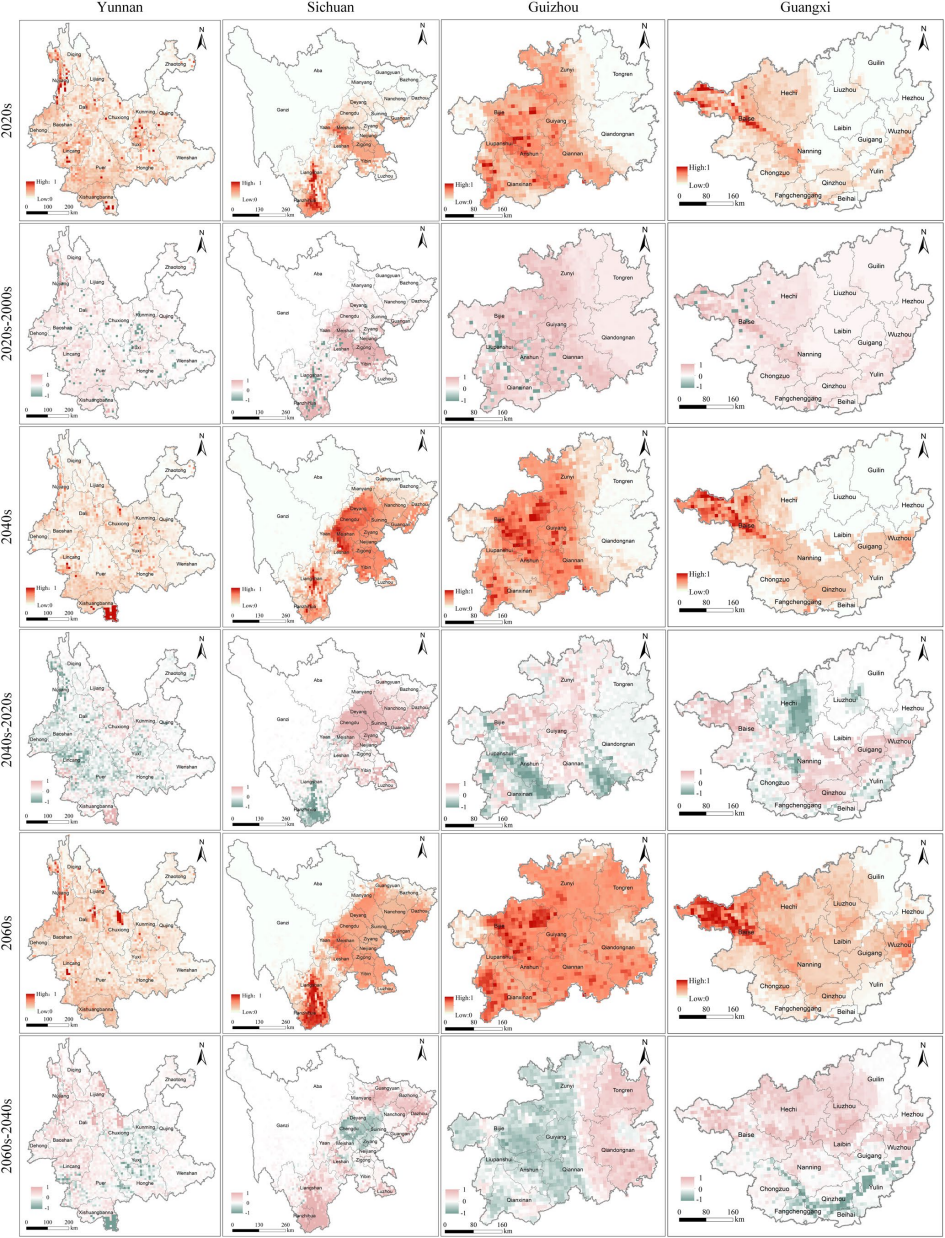
Predicted invasion risk and changes for *A. adenophora* in Yunnan, Sichuan, Guizhou, and Guangxi under SSP 3–7.0 climate scenario (2021–2080). It illustrates the predicted invasion risk for *A. adenophora* in Yunnan, Sichuan, Guizhou, and Guangxi under the SSP 3–7.0 climate scenario for the future periods 2021–2040, 2041–2060, and 2061–2080, as well as the changes between these periods. The first, third, and fifth rows show the predicted invasion risk, while the second, fourth, and sixth rows depict the changes compared to the previous period.

In four key provinces—Yunnan, Sichuan, Guizhou, and Guangxi (Figure 5), under the SSP3‐7.0 scenario, short‐term projections for the 2020s indicate that in all four provinces, the central and northeastern regions exhibit the most favorable conditions for the species' spread, while the western and southern parts show lower levels of risk. Sichuan and Guangxi have moderate levels of invasion risk. Compared to the current distribution, all four provinces experience significant increases in invasion risk. Mid‐term projections for the 2040s suggest that Yunnan remains a dominant invasion risk, with Sichuan and Guizhou showing notable increases, and Guangxi continues to expand northward. Comparing this period to the 2020s, the most significant increases occur in higher‐altitude areas of Sichuan and Guizhou, while the invasion risk is reduced in most parts of Yunnan. Long‐term projections for the 2060s indicate extensive areas of high invasion risk across all four provinces. Compared to the 2040s, the risk of invasion is expected to rise in certain areas, particularly in the northern and central parts of Yunnan, eastern and southeastern parts of Sichuan, central and northern regions of Guizhou, and northern and eastern areas of Guangxi. Conversely, the southern and western regions of Yunnan, the northwestern part of Sichuan, the southern areas of Guizhou, and the southern and southwestern parts of Guangxi show a decrease in risk. Compared to the SSP3‐7.0 scenario, under SSP2‐4.5, we predict fewer high‐risk areas across the four provinces (Figure S7), whereas, under SSP5‐8.5, a more significant extent of the high‐risk regions is likely to emerge (Figure S8).

## 4 Discussion

This study presents an integrated analysis of the invasion risk of *A. adenophora* in China, incorporating both climate and anthropogenic factors within the CMIP6 climate framework. Unlike previous research that predominantly relied on national‐scale models, our approach emphasizes region‐specific insights vital for effective management strategies. This approach aligns with the growing trend in ecological research to use detailed models for invasive species under climate change scenarios (Bradley et al. 2019; Early et al. 2016; Gallardo et al. 2016).

### 4.1 The Role of Climate and Human Activities in Driving Invasion Risk

Our findings highlight the intricate interplay between climatic and anthropogenic factors driving the invasion risk of *A. adenophora* , with notable regional variations in critical determinants. At the national level, population density and temperature‐related variables emerged as primary factors, consistent with other studies indicating that human activity and climate conditions significantly facilitate the invasion of *A. adenophora* (Gu et al. 2021; Tu et al. 2021). High population density, often linked with urban expansion, agriculture, and infrastructure development, alters native habitats and disrupts ecosystem balance, creating favorable conditions for invasive species (Gaertner et al. 2017). Temperature‐related variables further support the species' establishment and spread, aligning with findings from other studies that show climate as a critical driver in the distribution of invasive plants, particularly in regions where human‐induced habitat modifications coincide with conducive climate conditions (Seebens et al. 2021).

However, provincial‐level analysis reveals that factors such as temperature seasonality and precipitation patterns interact uniquely with human influences in each region, resulting in different impacts on the invasion of *A. adenophora* . For example, in Yunnan, the invasion of *A. adenophora* is significantly influenced by agricultural expansion and high population density; the combination of dense human populations and extensive cropland areas creates favorable conditions for its invasion and establishment (C. Wang et al. 2023). In contrast, precipitation patterns are the primary factor in Guizhou, highlighting how the region's humid and highly seasonal rainfall creates ideal conditions for the invasion of *A. adenophora* . This regional variability underscores a limitation in broad‐scale models, which may fail to capture the nuanced interactions between environmental and anthropogenic factors at finer scales. This study thereby supports the value of a fine‐grained, region‐specific approach, aligning with previous research underscoring the importance of local environmental and human drivers in determining invasion success (Early and Sax 2014; Gallardo et al. 2016; Seebens et al. 2021; Václavík and Meentemeyer 2012).

### 4.2 Shifts in Invasion Risk Under Future Scenarios

Projections based on CMIP6 scenarios indicate a northward and upward shift in *A. adenophora's* invasion risk by the 2060s, driven by warming temperatures and altered precipitation patterns (Tu et al. 2021). These shifts indicate a potential emergence of new hotspots in marginal central Chinese and high‐altitude regions. This trend aligns with observed patterns in other invasive species, where climate change facilitates expansion into novel ecological niches (Liu et al. 2020; Xian et al. 2023). Notably, the model forecasts a potential decline in invasion in southern regions like Yunnan and Guangxi due to “oversaturation,” where extreme climatic conditions surpass optimal thresholds for the species, a phenomenon also observed in tropical invasive plants (Allen and Bradley 2016; Bellard et al. 2012).

These findings highlight climate change's complex, non‐linear impacts on the distribution of invasive species, as global warming may expand invasion risk in some areas while reducing suitability in others (Bellard et al. 2012; Seebens et al. 2021). Recognizing this trend is crucial for adaptive management, as it indicates that invasion risks may not only spread to new areas but also recede from traditionally favorable zones. This highlights the importance of continuous monitoring and the need for dynamic management strategies that adapt to shifting environmental conditions.

### 4.3 Management Implications and Strategic Recommendations

Given the nuanced understanding of regional invasion dynamics gained from this study, we recommend that management efforts adopt a proactive, region‐specific approach. Our findings suggest prioritizing monitoring and control strategies in central China, northern Sichuan, and high‐altitude regions, emerging as high‐risk zones (Xian et al. 2022). Furthermore, implementing buffer zones in high‐risk areas can mitigate the spread by creating a barrier against invasion (Simberloff et al. 2013). Given the strong influence of human activity, policy interventions should aim to limit ecological disturbances in vulnerable areas by regulating land‐use changes, reducing habitat fragmentation, and promoting the restoration of native vegetation (Haddad et al. 2015).

### 4.4 Future Research Directions

Despite these advancements, several limitations remain, particularly regarding the impact of human activities such as urban expansion and infrastructure development. Future research should incorporate high‐resolution human activity data, including economic activity indices and road density, to improve our understanding of how anthropogenic changes intersect with climate factors over time. Additionally, exploring ensemble modeling approaches that combine multiple algorithms (e.g., RF, Gradient Boosting, and Neural Networks) may further enhance predictive robustness and accuracy (Fang et al. 2021). This multi‐model approach would advance current research by capturing non‐linear interactions between climate and human factors, offering a more comprehensive understanding of invasion dynamics.

In conclusion, this study highlights the importance of incorporating climate and anthropogenic factors into region‐specific models for predicting invasive species. Our findings offer a detailed perspective on invasion dynamics across China's diverse regions, emphasizing the need for targeted management strategies. These insights lay a foundation for adaptive management strategies that address unique regional vulnerabilities, supporting more targeted and effective responses to mitigate the ecological and economic impacts of *A. adenophora* . This regionally tailored approach enhances the scientific rigor of invasion modeling and contributes to practical, on‐the‐ground applications in controlling invasive species in complex and varied landscapes.

## Author Contributions

**Xiaolan Xie:** conceptualization (equal), formal analysis (equal), methodology (equal), writing – original draft (equal). **Tian Ma:** conceptualization (equal), visualization (equal), writing – review and editing (equal). **Yu Chen:** software (equal), writing – review and editing (equal). **Jun Zhuo:** writing – review and editing (equal). **Shuai Chen:** methodology (equal), visualization (equal), writing – review and editing (equal). **Tingting Kang:** investigation (equal), writing – review and editing (equal). **Mengmeng Hao:** writing – review and editing (equal). **Fangyu Ding:** writing – review and editing (equal). **Dong Jiang:** conceptualization (equal), project administration (equal), supervision (equal), writing – review and editing (equal).

## Conflicts of Interest

The authors declare no conflicts of interest.

## Supporting information


**Data S1:** ece372392‐sup‐0001‐Supinfo.pdf.

## Data Availability

The data and code have been successfully uploaded to the following 4TU. ResearchData repository: https://data.4tu.nl/private_datasets/SmUDpLrt9fUFxVZD1cBrNQJiDPyVKfI7ZTO8xUa1NJE.
