# Supplementary material for: Modeling the Potential Invasion Risk of Ageratina adenophora in China From an Ecological Suitability Perspective
Source: Ecol Evol. 2025 Oct 22;15(10):e72392. doi: 10.1002/ece3.72392 (PMC12541358; doi:10.1002/ece3.72392)
Supplement: Supplementary file 1 — Data S1: ece372392‐sup‐0001‐Supinfo.pdf. [file ECE3-15-e72392-s001.pdf]

Supplementary Information for:

Modeling the potential invasion risk of *Ageratina adenophora* in China from an ecological suitability perspective

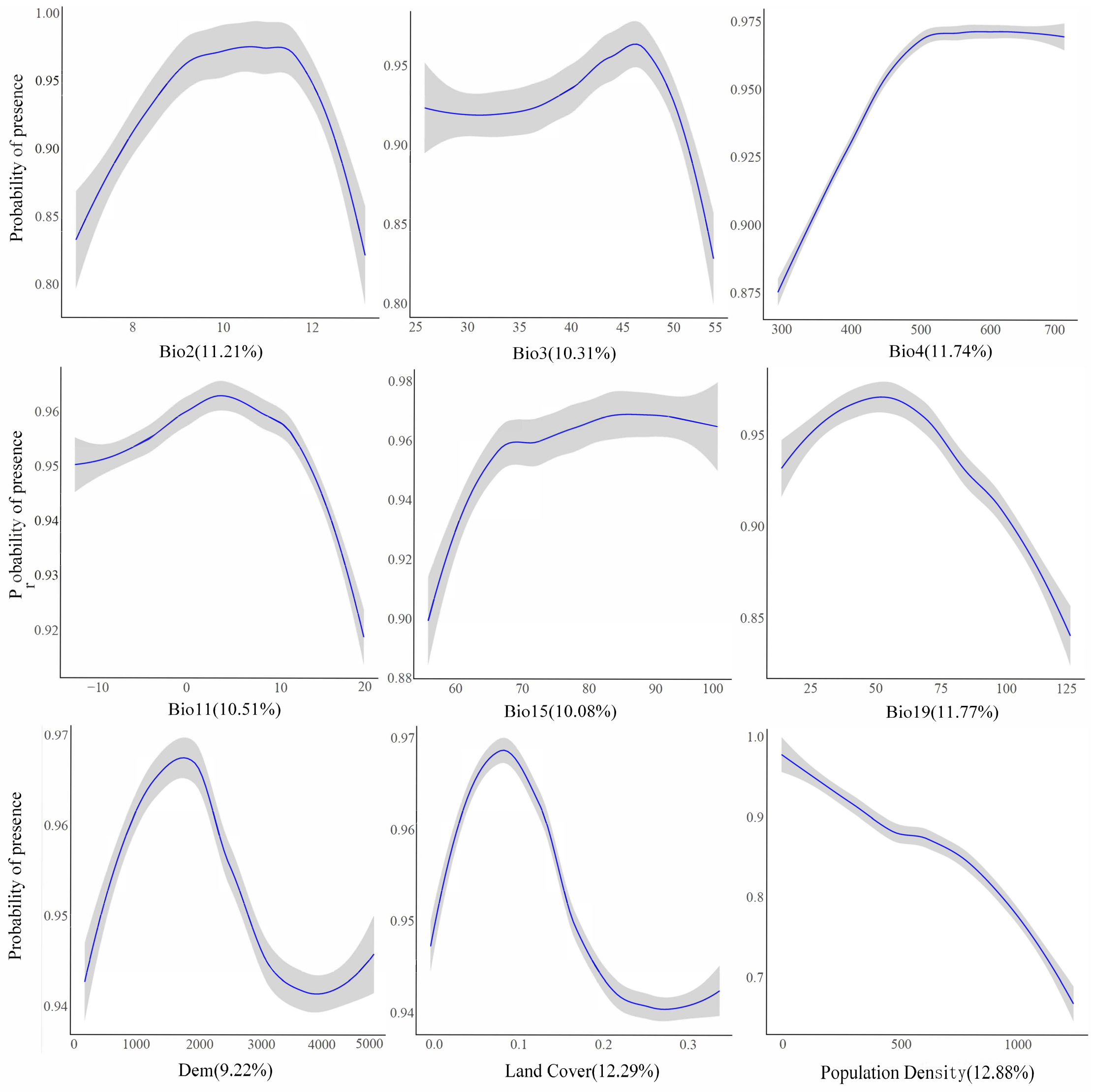

Figure S1. Partial dependence plots of factors and the probability of ecological suitability for *A. adenophora* in Yunnan. Each panel represents the relationship between a specific factor and the probability of the presence of the species. Each panel represents the relationship between a specific factor and the likelihood of the invasion of the species. The blue line indicates the estimated effect of the variable on habitat suitability, while the shaded gray areas represent the confidence intervals. The percentage in parentheses represents the contribution of each variable to the overall model.

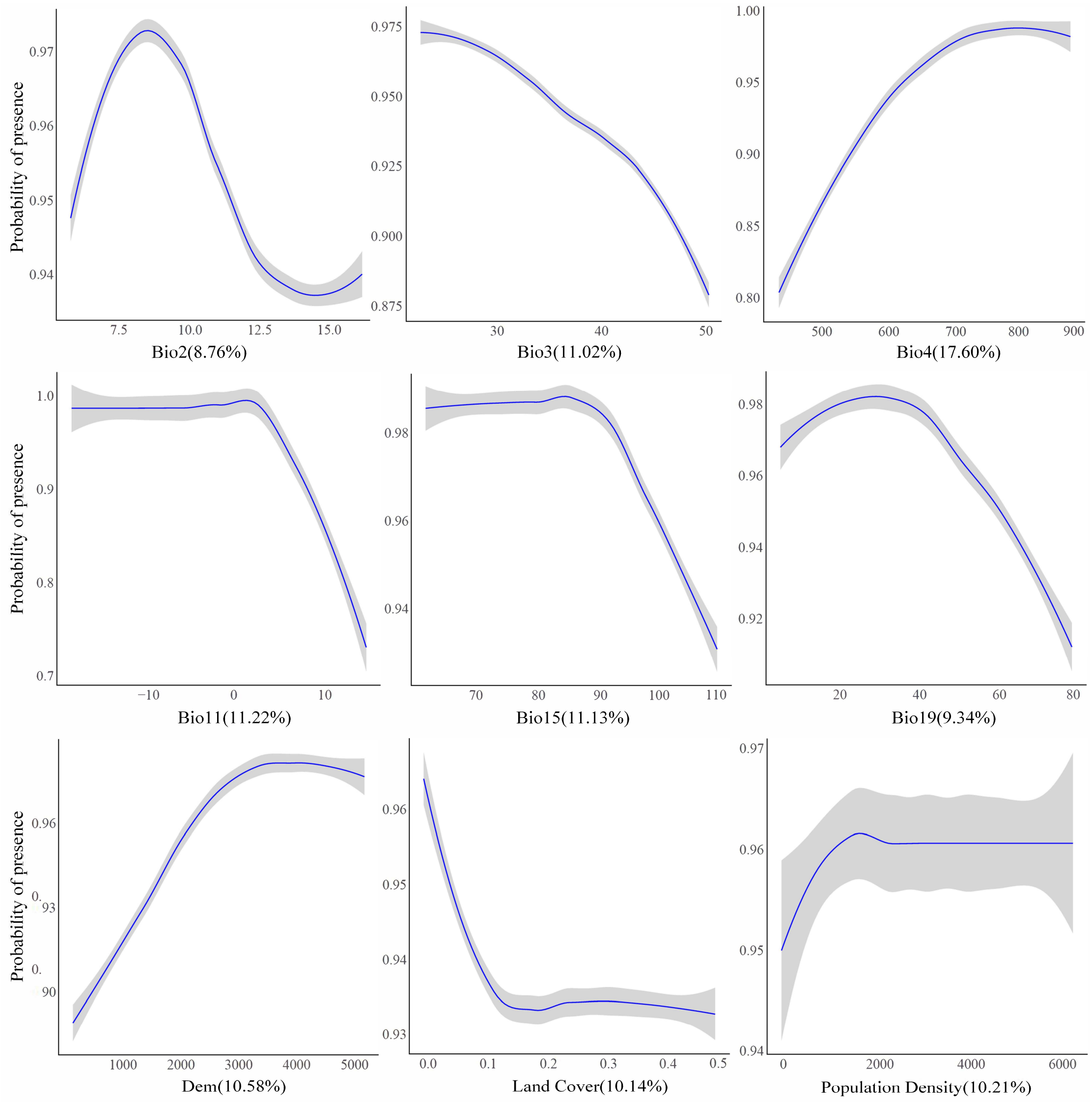

Figure S2. Partial dependence plots of environmental factors and the probability of ecological suitability for *A. adenophora* in Sichuan. Each panel represents the relationship between a specific factor and the probability of the presence of the species. Each panel represents the relationship between a specific factor and the likelihood of the invasion of the species. The blue line indicates the estimated effect of the variable on habitat suitability, while the shaded gray areas represent the confidence intervals. The percentage in parentheses represents the contribution of each variable to the overall model.

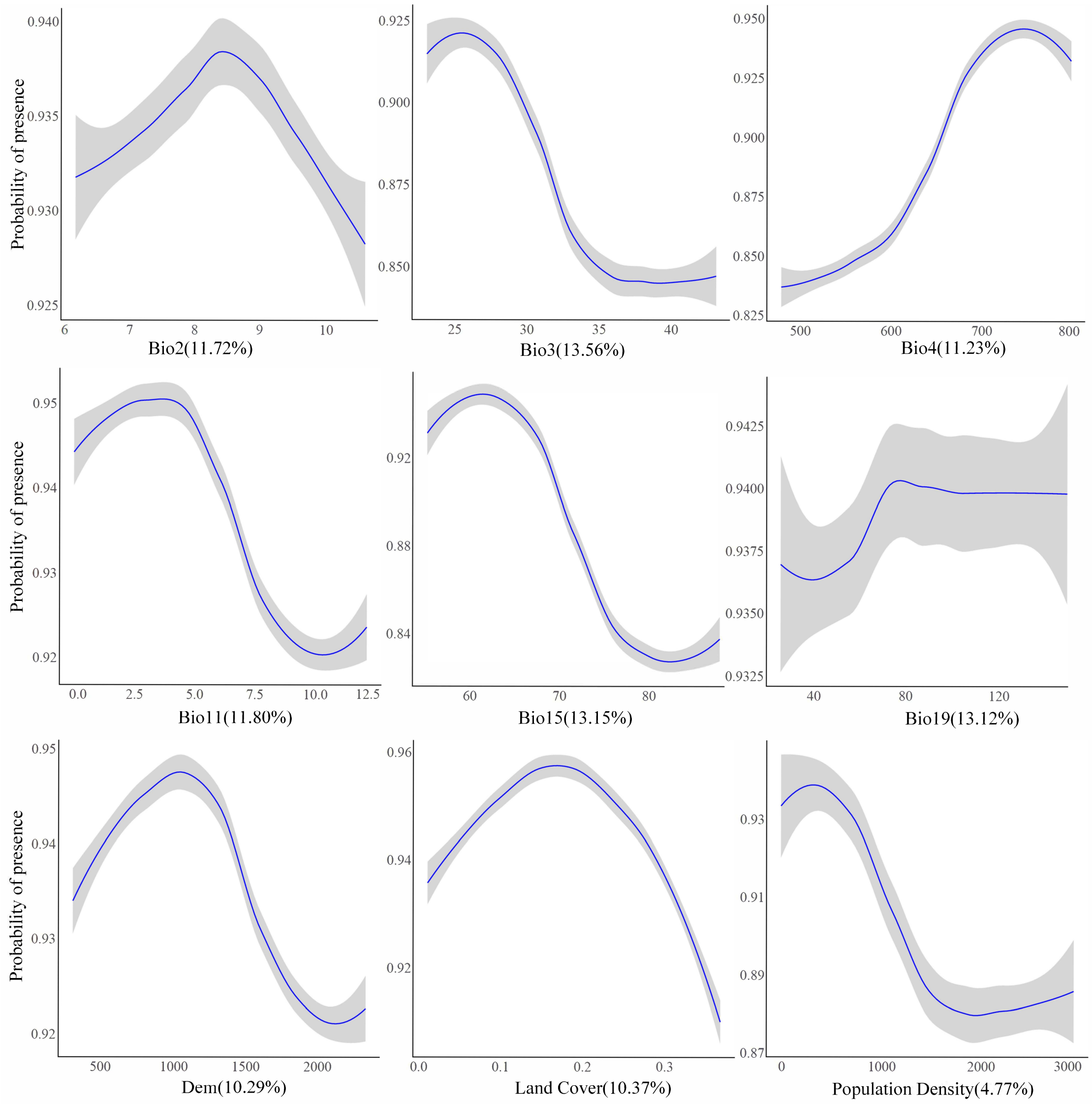

Figure S3. Partial dependence plots of environmental factors and the probability of ecological suitability for *A. adenophora* in Guizhou. Each panel represents the relationship between a specific factor and the probability of the presence of the species. Each panel represents the relationship between a specific factor and the likelihood of the invasion of the species. The blue line indicates the estimated effect of the variable on habitat suitability, while the shaded gray areas represent the confidence intervals. The percentage in parentheses represents the contribution of each variable to the overall model.

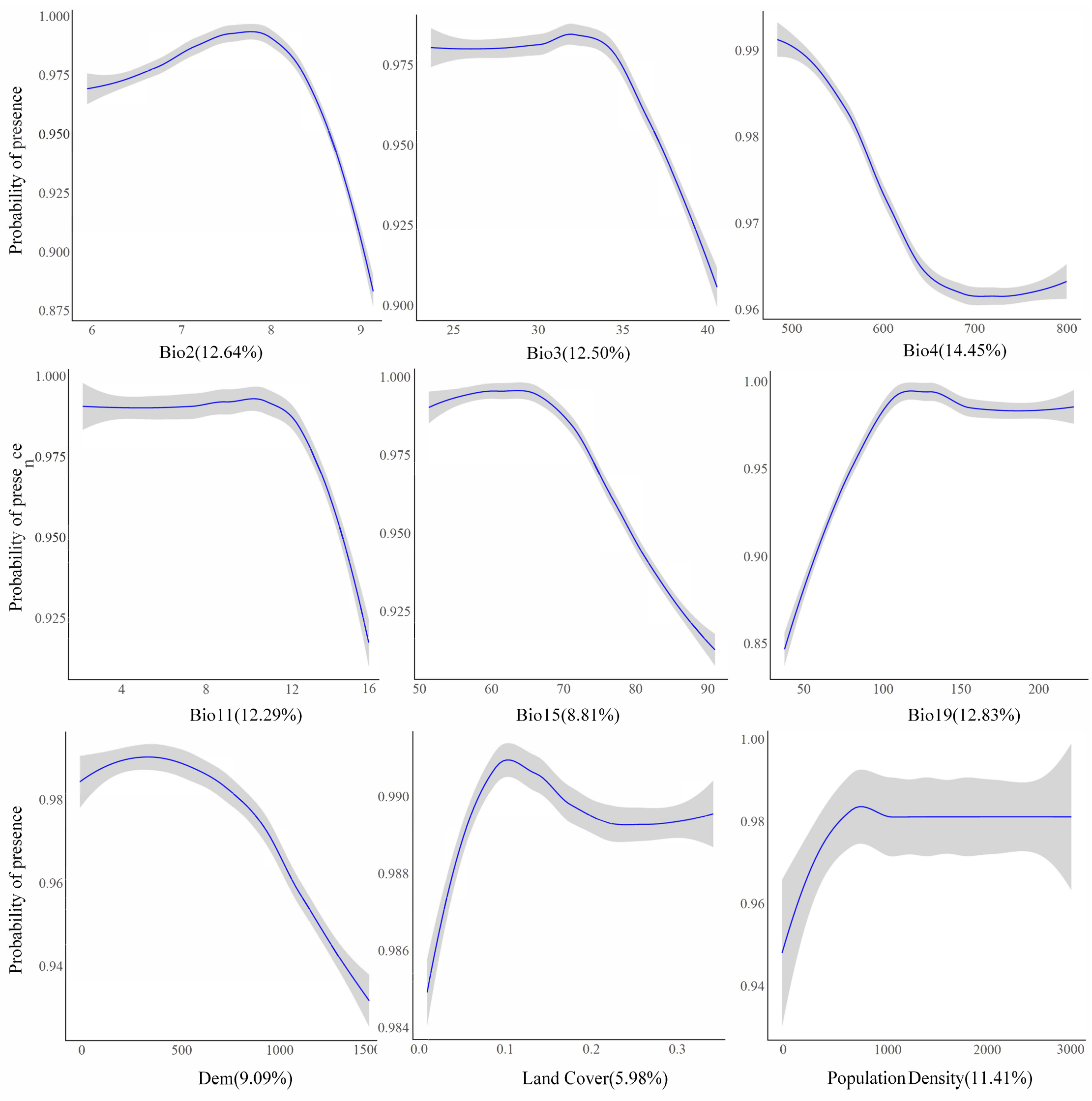

Figure S4. Partial dependence plots of environmental factors and the probability of ecological suitability for *A. adenophora* in Guangxi. Each panel represents the relationship between a specific factor and the probability of the presence of the species. Each panel represents the relationship between a specific factor and the likelihood of the invasion of the species. The blue line indicates the estimated effect of the variable on habitat suitability, while the shaded gray areas represent the confidence intervals. The percentage in parentheses represents the contribution of each variable to the overall model.

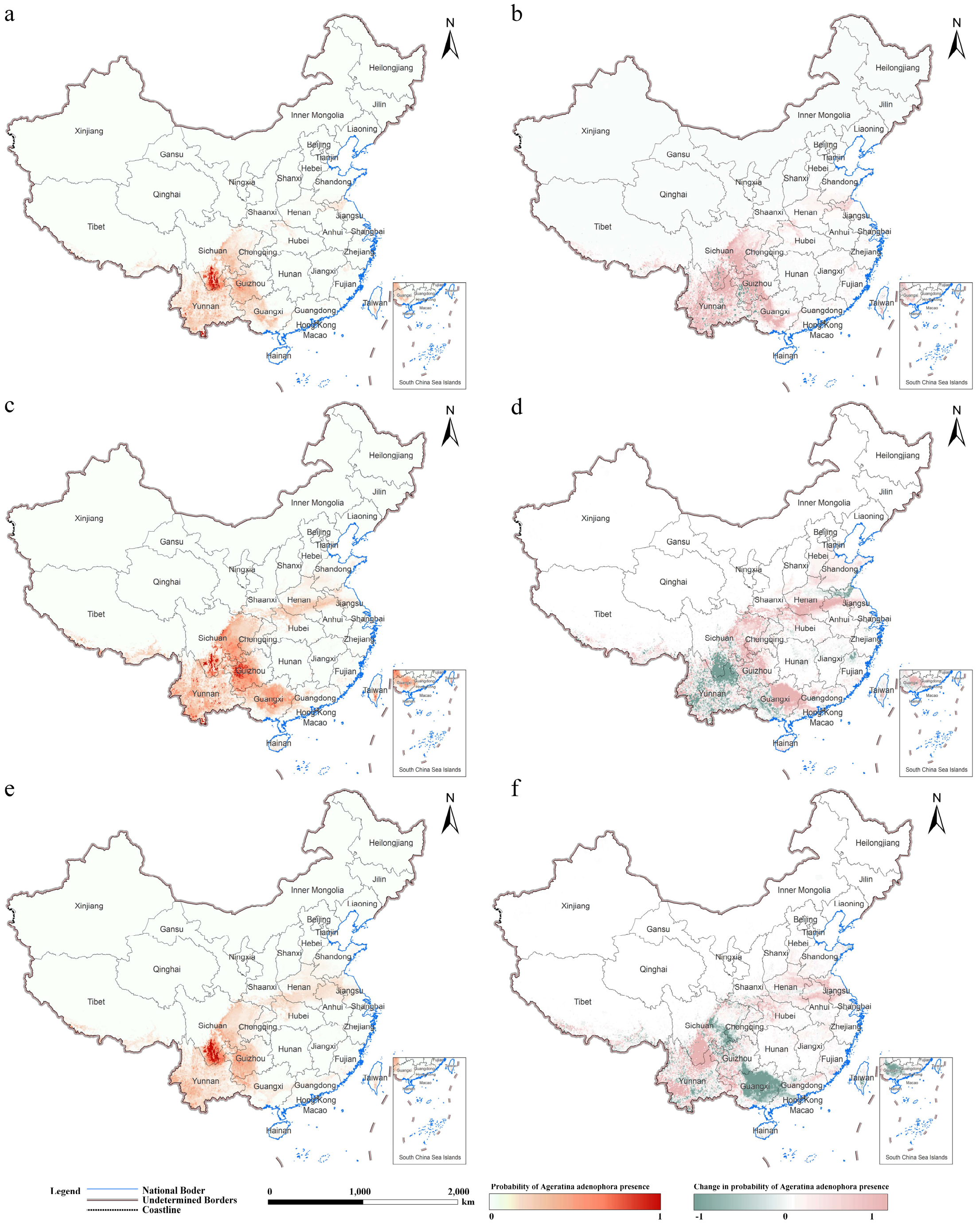

Figure S5. Predicted invasion risk and changes for *A. adenophora* under SSP 2-4.5 climate and human activity scenario (2021-2080). The left column (a, c, e) shows the predicted probability of invasion risk for the species in each period, with deeper red colors indicating higher probabilities. The right column (b, d, f) shows the changes in predicted invasion risk: (b) represents changes from the current period to 2021-2040, (d) shows changes from 2021-2040 to 2041-2060, and (f) indicates changes from 2041-2060 to 2061-2080.

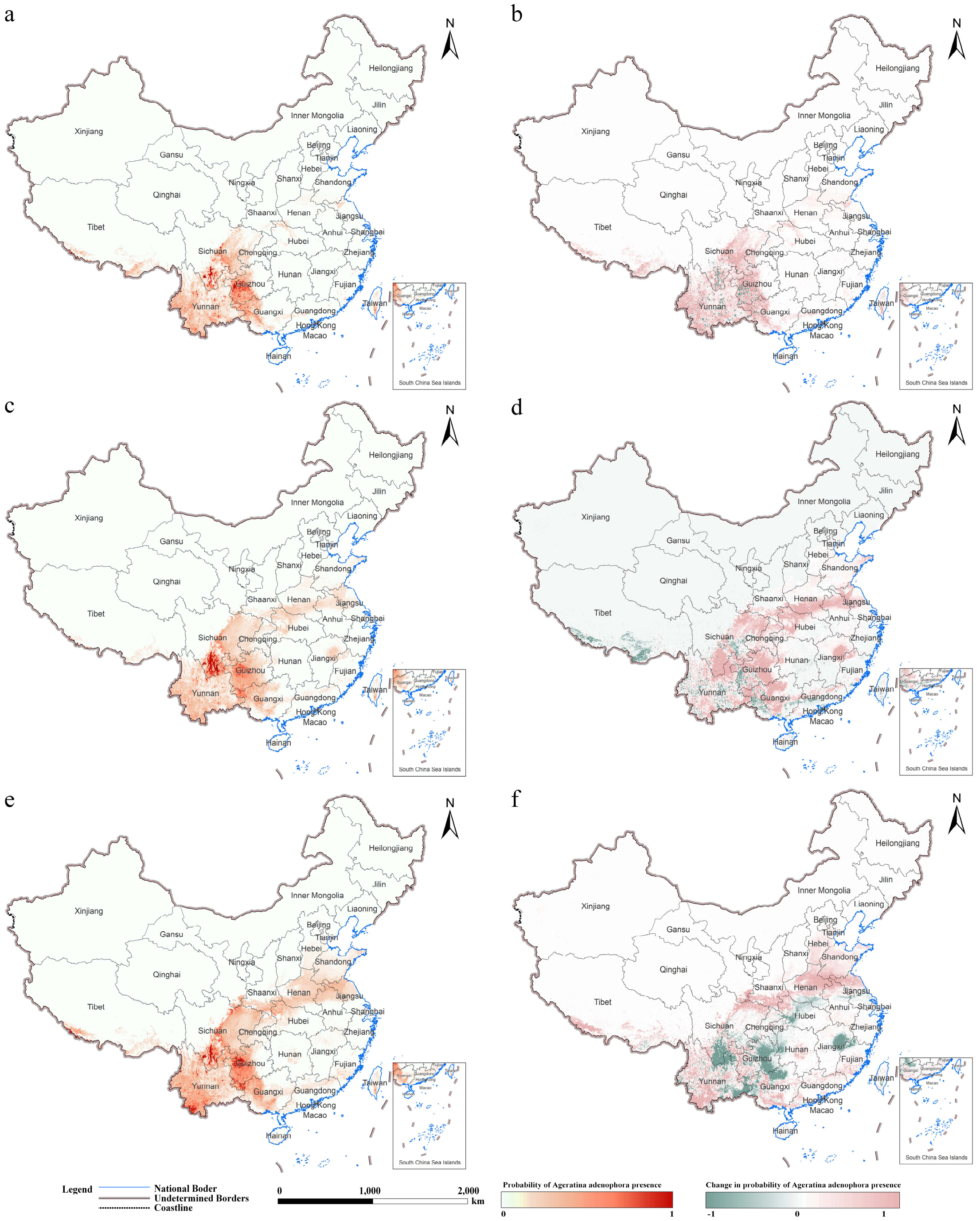

Figure S6. Predicted invasion risk and changes for *A. adenophora* under SSP 5-8.5 climate and human activity scenario (2021-2080). The left column (a, c, e) shows the predicted probability of invasion risk for the species in each period, with deeper red colors indicating higher probabilities. The right column (b, d, f) shows the changes in predicted invasion risk: (b) represents changes from the current period to 2021-2040, (d) shows changes from 2021-2040 to 2041-2060, and (f) indicates changes from 2041-2060 to 2061-2080.

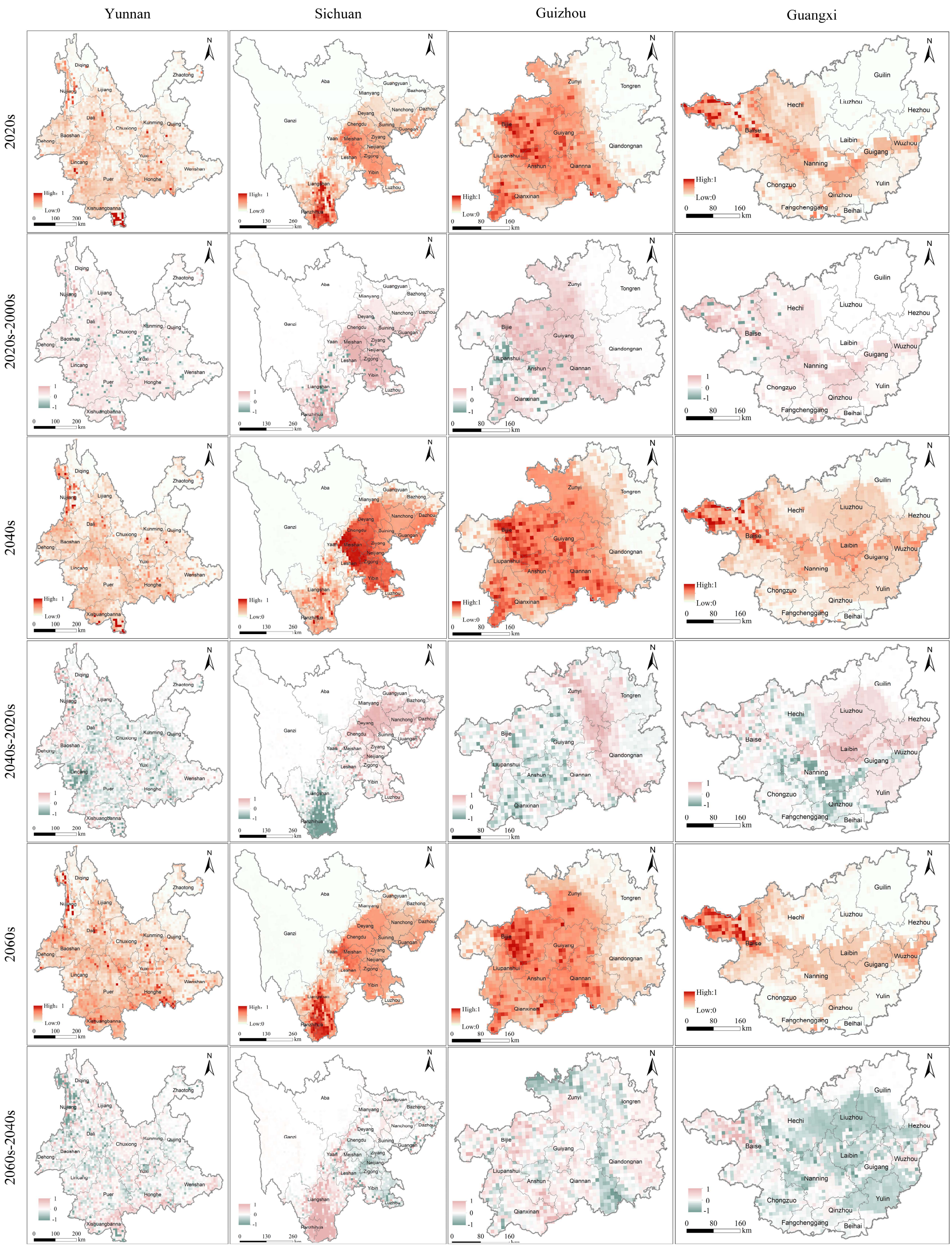

Figure S7. Predicted invasion risk and changes for *A. adenophora* in Yunnan, Sichuan, Guizhou, and Guangxi under SSP 2-4.5 climate scenario (2021-2080). It illustrates the predicted invasion risk for *A. adenophora* in Yunnan, Sichuan, Guizhou, and Guangxi under the SSP 2-4.5 climate scenario for the future periods 2021-2040, 2041-2060, and 2061-2080, as well as the changes between these periods. The first, third, and fifth rows show the predicted invasion risk, while the second, fourth, and sixth rows depict the changes compared to the previous period.

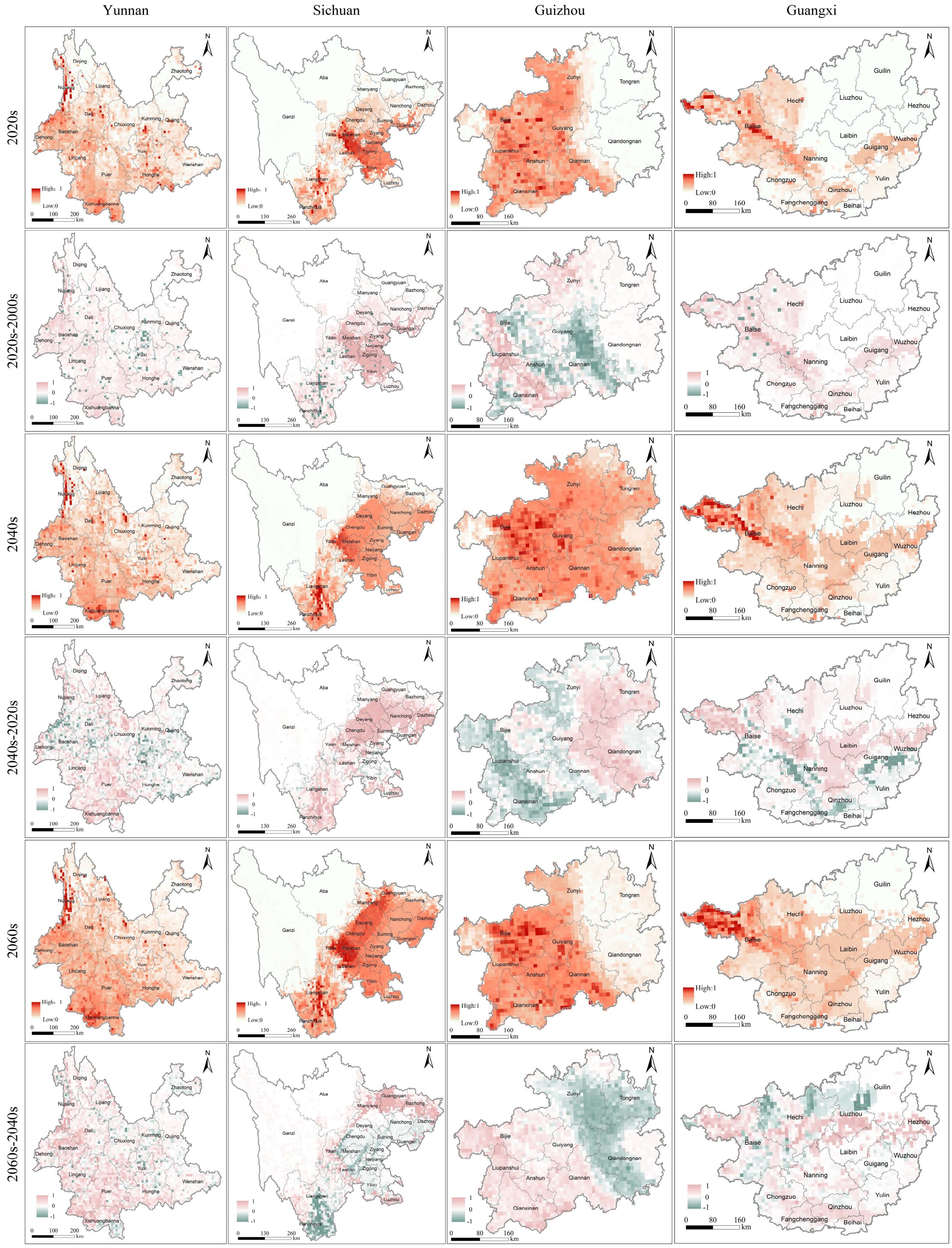

Figure S8. Predicted invasion risk and changes for *A. adenophora* in Yunnan, Sichuan, Guizhou, and Guangxi under SSP 5-8.5 climate scenario (2021-2080). It illustrates the predicted invasion risk for *A. adenophora* in Yunnan, Sichuan, Guizhou, and Guangxi under the SSP 5-8.5 climate scenario for the future periods 2021-2040, 2041-2060, and 2061-2080, as well as the changes between these periods. The first, third, and fifth rows show the predicted invasion risk, while the second, fourth, and sixth rows depict the changes compared to the previous period.

Table S1. The variables used in this study.

Variables	Description
Bio1	Annual mean temperature
Bio2	Mean diurnal range (Mean of monthly (max temp - min temp))
Bio3	Isothermality (BIO2/BIO7) (×100)
Bio4	Temperature seasonality (standard deviation ×100)
Bio5	Max temperature of warmest month
Bio6	Min temperature of coldest month
Bio7	Temperature annual range (Bio5-Bio6)
Bio8	Mean temperature of wettest quarter
Bio9	Mean temperature of driest quarter
Bio10	Mean temperature of warmest quarter
Bio11	Mean temperature of coldest quarter
Bio12	Annual precipitation
Bio13	Precipitation of wettest month
Bio14	Precipitation of driest month
Bio15	Precipitation seasonality (coefficient of variation)
Bio16	Precipitation of wettest quarter
Bio17	Precipitation of driest quarter
Bio18	Precipitation of warmest quarter
Bio19	Precipitation of coldest quarter
DEM	digital elevation model
Population Density	The number of people living per unit of area
Land Cover	Proportion of agricultural land

Table S2. The relative contribution of covariates to the distribution of *A. adenophora* in China.

Variables	Relative contribution ± Standard Deviation, %
**Environmental variables†**	**76.18**
Bio4	12.92±1.15
Bio3	11.83±1.00
Bio15	11.80±1.06
Bio19	10.78±1.03
DEM	10.50±0.90
Bio11	9.81±0.76
Bio2	8.54±1.46
**Human activities†**	**23.82**
Population density	14.92±1.83
Land cover	8.90±1.63

Note: †Sum of relative contribution for each category.

Table S3. The relative contribution of covariates to the distribution of *A. adenophora* in Yunnan.

Variables	Relative contribution ± Standard Deviation, %
**Environmental variables†**	**74.83**
Bio19	11.77±0.84
Bio4	11.74±0.53
Bio2	11.21±0.63
Bio11	10.51±0.60
Bio3	10.31±0.48
Bio15	10.08±0.66
DEM	9.22±0.57
**Human activities†**	**25.17**
Population density	12.88±1.09
Land cover	12.29±0.86

Note: †Sum of relative contribution for each category.

Table S4. The relative contribution of covariates to the distribution of *A. adenophora* in Sichuan.

Variables	Relative contribution ± Standard Deviation, %
**Environmental variables†**	**77.65**
Bio4	17.60±0.71
Bio11	11.22±0.87
Bio15	11.13±0.79
Bio3	11.02±0.50
DEM	10.58±0.53
Bio19	9.34±0.52
Bio2	8.76±0.42
**Human activities†**	**22.35**
Population density	10.21±1.12
Land cover	12.14±0.59

Note: †Sum of relative contribution for each category.

Table S5. The relative contribution of covariates to the distribution of *A. adenophora* in Guizhou.

Variables	Relative contribution ± Standard Deviation, %
**Environmental variables†**	**84.86**
Bio3	13.56±0.77
Bio15	13.15±0.66
Bio19	13.11±1.03
Bio11	11.80±0.97
Bio2	11.72±1.09
Bio4	11.23±0.55
DEM	10.29±0.87
**Human activities†**	**15.14**
Land cover	10.37±0.91
Population density	4.77±1.15

Note: †Sum of relative contribution for each category.

Table S6. The relative contribution of covariates to the distribution of *A. adenophora* in Guangxi.

Variables	Relative contribution ± Standard Deviation, %
**Environmental variables†**	**82.61**
Bio4	14.45±1.05
Bio19	12.83±0.72
Bio2	12.64±1.28
Bio3	12.50±0.87
Bio11	12.29±0.71
DEM	9.09±0.94
Bio15	8.81±0.67
**Human activities†**	**17.39**
Population density	11.41±1.14
Land cover	5.98±0.80

Note: †Sum of relative contribution for each category.
